# Supplementary material for: Nasal microbionts differentially colonize and elicit cytokines in human nasal epithelial organoids
Source: mSphere. 2025 Sep 30;10(10):e00493-25. doi: 10.1128/msphere.00493-25 (PMC12570476; doi:10.1128/msphere.00493-25)
Supplement: Figure S1 — Experiments at 37°C and cells/transwell. [file msphere.00493-25-s0001.pdf]

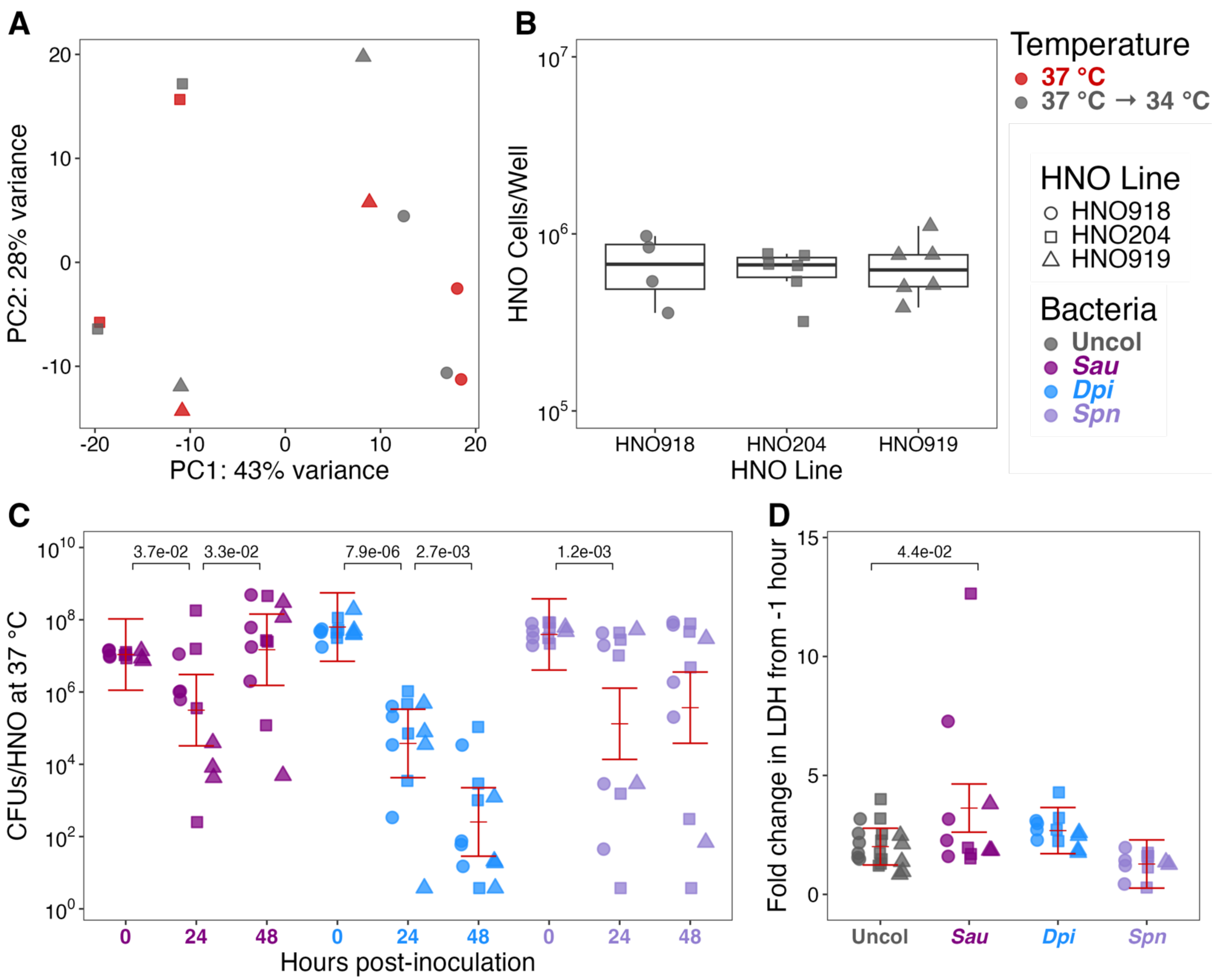

Figure S1. **Nasal microbionts colonize HNOs at human internal body temperature, 37 °C.** (**A**) In a Principal Component Analysis, epithelial transcription (read counts) within an HNO line was comparable between HNOs differentiated at 37 °C for 21 days (red) and HNOs shifted to 34 °C (gray) for an additional 2 days after the 21 days of differentiation at 37 °C (*n* = 2 independent experiments with 3 HNO lines). (**B**) HNO lines derived from different donors had comparable cells per transwell. The median (range) number of cells in each HNO was 6.9 x 10^5 for line HNO918 (3.58 x 10^5 – 9.72 x 10^5), 6.68 x 10^5 (3.2 x 10^5 – 7.72 x 10^5) for line HNO204, and 6.37 x 10^5 for line HNO919 (3.84 x 10^5 – 1.1 x 10^6) in *n* = 4 for HNO918, *n* = 6 for HNO204, and *n* = 6 in HNO919. (**C**) HNOs were monocolonized with *S. aureus* (purple), *D. pigrum* (blue), and *S. pneumoniae* (lavender) at 37 °C for up to 48 h. At time 0, 10^7 CFUs of a bacterium in 15 µL of EBSS were inoculated apically. Recovered CFUs/HNO at 24

and 48 h are shown. (**D**) Fold change in lactate dehydrogenase release (LDH) of uncolonized (gray) HNOs and of HNOs colonized with *S. aureus* (purple), *D. pigrum* (blue), or *S. pneumoniae* (lavender) into HNO basal medium at 48 h compared to -1 h samples from the same well at 37 °C. HNOs colonized by *S. aureus* had 1.8-fold higher basal LDH release compared to the uncolonized control. For **C** and **D,** the independent experiments per HNO line were HNO918 $\geq$ 4, HNO204 $\geq$ 4, and HNO919 $\geq$ 2. Data (**C, D**) were analyzed using a LMM (**Tables S1A-B)** to determine statistical significance and the Holm method was used to adjust *p*-values (shown above the horizontal bars) for multiple comparisons (0 to 24 h and 24 to 48 h in C and uncolonized to each bacterial treatment in D). Vertical brackets represent the model-predicted mean values and confidence intervals (+/- twice the standard error of the mean).
