# Supplementary material for: Nasal microbionts differentially colonize and elicit cytokines in human nasal epithelial organoids
Source: mSphere. 2025 Sep 30;10(10):e00493-25. doi: 10.1128/msphere.00493-25 (PMC12570476; doi:10.1128/msphere.00493-25)
Supplement: Figure S2 — HEK-Blue IL-1R cell assay. [file msphere.00493-25-s0002.pdf]

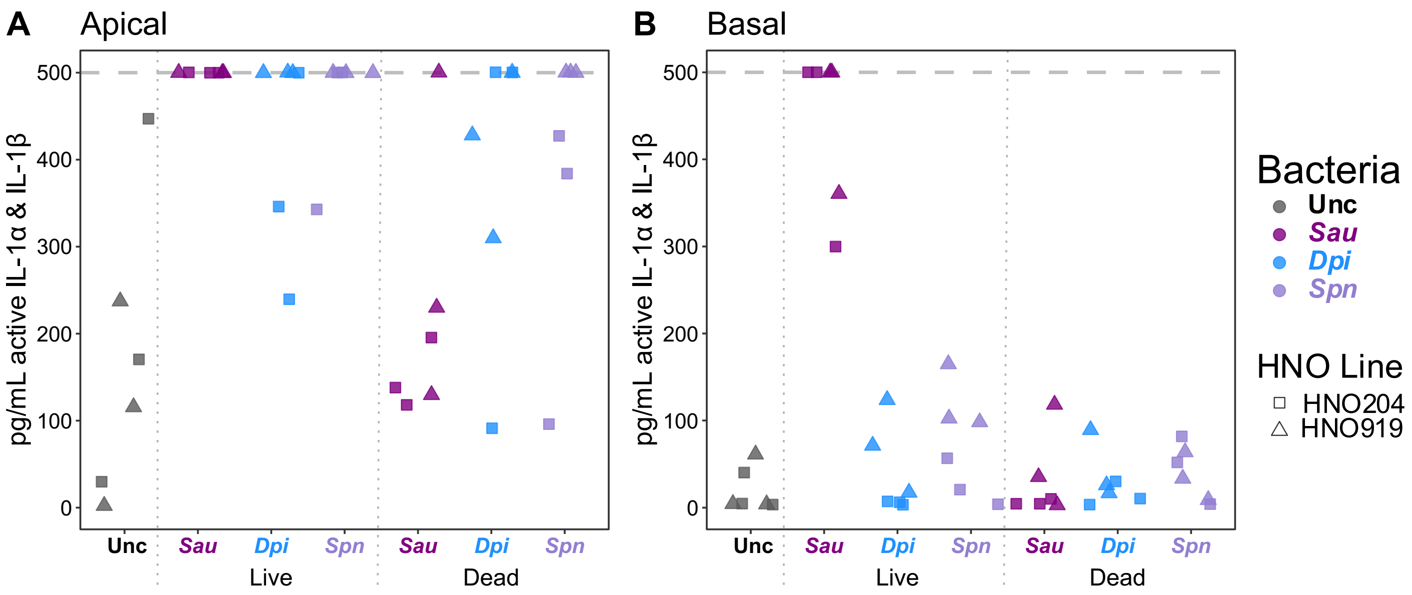

Figure S2. **In response to live *S. aureus* monocolonization, HNO production of IL-1α activity dominates over IL-1RN activity resulting in IL-1 receptor activation apically and basally.** (**A**) The apical and (**B**) basal IL-1α and IL-1β detected in the cytokine assays in Figure 5A are active. HEK-Blue™ IL-1R Cells were used to detect catalytically active IL-1α and IL-1β produced by HNOs (without distinction) to the extent that these were greater than IL-1RN activity. The upper limit of detection of the assay was 500 pg/mL (gray dashed line). For A, B, and C, data are from three independent experiments in each of two HNO lines (HNO204, HNO919). HEK-Blue™ IL-1R Cells (InvivoGen HKB-IL1R) were propagated and assays were performed according to the manufacturer's instructions. Briefly, after adding 20 µL of either HNO basal medium or apical wash to the bottom of a 96-well plate, we inoculated each well with 3 x 10^5 HEK-Blue cells and incubated for 22 hours at 37 °C in a humidified 5% CO_2 incubator. HEK-Blue cells partially adhere to the bottom of the 96-well plate during incubation. At collection, plates were tapped gently to mix the liquid and 20 µL of spent HEK medium from each well was transferred into a corresponding well of a new 96-well plate prior to addition of 180 µL of freshly prepared QUANTI-Blue™ Solution (Invivogen REPQBS) per well. The assay developed at 37 °C for 2.5 hours before reading absorbance at 620 nm. A standard curve was constructed using InvivoGen recombinant human IL-1β as a positive control. Detailed code to analyze the HEK-Blue data and create the plots is available online (https://klemonlab.github.io/HNOBac_Manuscript/Methods_HekBlue.html).
